# Pharmacokinetics of Human Red Blood Cell Microparticles Prepared Using High-Pressure Extrusion Method

**DOI:** 10.3389/fphar.2018.00599

**Published:** 2018-06-11

**Authors:** Wenche Jy, Ashish K. Rehni, Carlos Bidot, Hever Navarro-Quero, Conner R. Haase, Sebastian Koch, Yeon S. Ahn, Kunjan R. Dave

**Affiliations:** ^1^Wallace H. Coulter Platelet Laboratory, Division of Hematology and Oncology, Department of Medicine, Miller School of Medicine, University of Miami, Miami, FL, United States; ^2^Cerebral Vascular Disease Research Laboratories, Miller School of Medicine, University of Miami, Miami, FL, United States; ^3^Department of Neurology, Miller School of Medicine, University of Miami, Miami, FL, United States; ^4^Neuroscience Program, Miller School of Medicine, University of Miami, Miami, FL, United States

**Keywords:** hemostasis, dose response, particle size, shelf-life, half-life, volume of distribution

## Abstract

Red blood cell microparticles (RMPs) is a high potency hemostatic agent, which may serve as a viable therapeutic approach. They generate thrombin *in vitro* and effective in arresting bleeding in animal bleeding models. However, prior to ascertaining the clinical efficacy of RMPs, detailed preclinical evaluation is necessary. Therefore, we aimed to characterize RMPs, ascertain their stability, and determine their pharmacokinetics in rats. RMPs were prepared from human RBCs by a high-pressure extrusion method. Pharmacokinetic parameters were computed from groups receiving various RMPs dosing regimens. Volume of distribution, elimination rate constant, and clearance for RMPs were also assessed. Major portion of prepared microparticles were RMPs and a very small portion of particles were from platelets and leukocytes. RMPs were stable when stored at 5 and -20°C for at least 12 months. *In vivo* half-life was found to vary for each paradigm, but in general, was less than 2 min for most of the paradigms evaluated. Our results demonstrate that RMPs are stable during prolonged storage and have a short half-life. Therefore, the clinical use of RMPs as a hemostatic agent, within a tailored treatment paradigm, may be advantageous in achieving prolonged systemic therapeutic benefit without provoking any thrombotic complications.

## Introduction

Cell derived microparticles (MPs) are small membrane vesicles that originate from platelets, endothelial cells, leukocytes, and red blood cells (RBCs), and naturally occur in low quantities in the blood (Burnier et al., 2009). During cell activation, apoptosis, and senescence, these vesicles bud from cell membranes and externalize phospholipids anchored to the inner leaflet of the lipid membrane (Burnier et al., 2009). One such exposed phospholipid, phosphatidylserine (PS), has been linked to the procoagulant properties associated with MPs (Tesse et al., 2006). Phosphatidylserine plays an active role in the coagulation cascade; when externalized, it triggers clotting factors to become activated (Leventis and Grinstein, 2010). Some microparticles also express tissue factor (TF), which triggers blood coagulation (Owens and Mackman, 2011). Due to these procoagulant effects, MPs have been investigated in the present study for their potential use as hemostatic agents.

In previous studies, MPs derived from red blood cells (RMPs) have been found to be positive for annexin V binding, a proxy for PS, without expressing tissue factor, thereby making these particles ideal for controlled clotting at the site of injury (Jy et al., 2013). In an *ex vivo* study on patients with platelet and coagulation disorders, RMPs decreased the lag time to initial fibrin formation (Jy et al., 2013). Additional experiments in thrombocytopenic animal models (rats and rabbits) showed RMPs to significantly shorten bleeding time (Jy et al., 2013). These results, in part, are attributed to the ability of RMPs to generate thrombin via the intrinsic pathway even in the absence of tissue factor (Jy et al., 2013; Rubin et al., 2013). In contrast to other hemostatic agents, the precursor of RMPs is abundant allowing for sufficient production, and because they are obtained from universal donor blood, these particles would minimize alloimmunization or toxic side-effects. RMPs additionally do not possess tissue factor, allowing them to cause thrombosis solely at the site of injury (Jy et al., 2013). Given that previously investigated treatments for life threatening hemorrhage, e.g., cerebral bleeding include recombinant activated factor VIIa, prothrombin complex concentrates, and platelet transfusions which increase the rate of adverse events or have limited improvement on functional outcomes (Mayer et al., 2008; Diringer et al., 2010; Yuan et al., 2010; Dowlatshahi et al., 2012; Baharoglu et al., 2016), there is a clear need for additional therapeutic interventions to treat intracranial hemorrhage (Sonni et al., 2014). To fill this demand, RMPs could be beneficial in the treatment of conditions such as ruptured cerebral aneurysms, hemorrhagic stroke, and brain trauma given the benefits of these particles including their availability, safety, and targeted action.

In addition to the hemostatic action of RMPs, the pharmacokinetics are important to investigate in order to evaluate the safety profile of these particles as a therapeutic agent. Size, purity, and stability are key factors in determining the suitability of RMPs. In order to control bleeding, these particles must travel through the small blood vessels without occluding these narrow vessels or capillaries, necessitating that the size of RMPs must be appropriate. It is also essential to confirm the purity and safety profile of RMPs produced by a method intended for mass production. It has been demonstrated that RMPs do not possess TF while microparticles from other cells present in blood do, thus establishing the purity of RMPs is essential (Jy et al., 2013). Although there are benefits and drawbacks to *in vivo* pharmacokinetic studies, they are essential before designing clinical studies (Saeidnia et al., 2015). Another goal of *in vivo* pharmacokinetic studies is to confirm that the agent is reaching therapeutic level without potential toxicity (Parasuraman, 2011). Considering the varying characteristics of bleeding in different conditions, it is also essential to evaluate many paradigms so that appropriate dosage regimens can be used for respective conditions.

In view of the previous research and the gap in knowledge, the goal of this study was to evaluate the stability, purity, and pharmacokinetics of RMPs in a rat model. All of these measures were evaluated with the aim to enable future efficacy and safety studies of RMPs.

## Materials and Methods

### Preparation of RMP

To obtain sufficient quantities of RMPs for experimentation, RMPs were produced by high-pressure extrusion of washed human red blood cells. Type O, Rh+ and leukoreduced packed RBCs were obtained from the OneBlood center located in Ft. Lauderdale, FL, United States (formally known as South Florida Community Blood Service). Each blood unit used passed every FDA-required test for human transfusion. RMPs were prepared within 14 days of blood withdrawal. The RBCs were washed twice with isotonic saline by centrifuging at 200 × g for 10 min. The resulting RBC pellets were resuspended in isotonic saline to make the final hematocrit close to 25%. The diluted RBC suspension was passed through a Constant System Cell Disruptor (Northants, United Kingdom) twice at 35,000 psi to yield RBC membrane fragments, i.e., RMPs. In this device, a sample is placed into a high pressure cylinder. Once the desired pressure is reached, a piston forces the sample through a fixed orifice at high velocity. The samples then impact on a cooled heat exchange surface before entering the sample collection outlet. This instrument had been used to lyse cells earlier (Hewinson et al., 2008). The resulting RMPs were washed twice with isotonic saline by centrifuging at 25,000 × *g* for 30 min. The resulting RMP pellets were re-suspended in isotonic saline to achieve ¼ of the original packed RBCs volume. The RMPs were aliquoted into small vials, lyophilized, sealed and stored at -80°C. For *in vitro* or *in vivo* experiments, the lyophilized RMPs were reconstituted using equal volume of distilled water. After vortexing for 30 s, the reconstituted RMPs were passed through a 2 μm syringe filter to remove RMP aggregates. The resulting RMPs were used for all experiments described in the present study.

### Protein Measurement

The protein content of RMPs was determined using Pierce BCA protein Assay Kit (Thermo Scientific, Weston, FL, United States). The BCA working reagent and protein standard were prepared by following the instructions provided by the manufacturer. Bovine serum albumin was used as a standard.

### Quantitation of RMPs

After reconstituting lyophilized RMPs and passing through 2 μm filters, the RMP samples were diluted 1:1000 with isotonic saline. 20 μL of diluted RMP sample and 4 μL of anti-CD235a-PE, anti-CD41-FITC, anti-CD45-PE or anti-CD62E were mixed, incubated on a shaker (60 rpm) for 20 min, further diluted with 976 μL of Phosphate Buffered Saline and were assayed using a flow cytometer. Prior to assay for RMP count, the flow cytometer (Beckman Coulter Model FC-500) was calibrated using Megamix (Stago) and CountBright (Thermo Scientific) beads. The former was used to adjust the voltages of photomultiplying tube (PMT) for forward scatter (FS), side scatter (SS), fluorescent parameter 1 (FL1), FL2, and the boundary of RMP distribution as per the manufacturer’s instructions. The latter was used for calibrating the flow rate. The discriminator (trigger) was set at FL1 = 1 or FL2 = 1 depending on the markers used. The run time was set to 1 min. The flow counts of the RMP region obtained from the assay were used for calculating the sample’s RMP concentration using the following formula: Concentration of RMP (counts/mL) = flow count × (1 mL/flow rate) × dilution factor.

### Purity

The degree of the purity of RMP samples was determined by measuring levels of leukocyte microparticles (LMP) (anti-CD45), platelet microparticles (PMP) (anti-CD41), and RMP (anti-CD235a) markers using flow cytometry. The purity of RMPs was calculated using the following formula: % Purity of RMP = [RMP concentration/(RMP concentration + LMP concentration + PMP concentration)] × 100.

### Size

The size distribution was determined using two independent methods. Size was measured at baseline using both methods, while size for the stability experiment was measured in samples stored at -20°C using the flow cytometric method only.

#### DELSA Method

Reconstituted filtered RMPs were shipped overnight to the Particle Characterization Laboratory, ImmunoSite Technologies, Miramar, FL on ice. Upon arrival the next day, samples were vortexed, filtered and diluted 1:11 with saline and vortexed again before injecting into the DelsaNano Submicron Particle Size and Zeta Potential Analyzer. The DELSA method determine the particle size by measuring the dynamic light scattering related to the Brownian motion of microscopic particles suspended in a fluid (BeckmanCoulter, 2018).

#### Flow Cytometric Method

This method is based on the findings that the size of a RMP (diameter) is proportional to the log FS amplitude. We measured the FS amplitude of 3 different sizes of beads (0.5, 0.9, and 3 mm) and found a linear correlation of log FS vs. log bead diameters. The size of RMPs was obtained by converting their log FS amplitude to diameter based on the standard curve of beads. The lower limit of size distribution by this method was 0.1 μm.

### Stability

For stability studies, lyophilized RMPs sealed in closed vials were shipped to the SciSafe biological and pharmaceutical storage facility on dry ice overnight. Upon receipt by SciSafe, the RMP samples were stored at -20°C, 5°C, 25°C/60% relative humidity (RH), 30°C/65% RH, or 40°C/75% RH. Upon completion of desired storage duration, samples were shipped back to the University of Miami on dry ice. The procoagulant activity, RMP counts, and size were then analyzed at 3 and 6-month intervals for all storage conditions; the -20, 5, and 25°C/60% conditions were additionally evaluated at 9 and 12-month intervals.

### Measurement of Procoagulant Activity Using Thromboelastography for Stability and *in Vitro* Dose Response Studies

The procoagulant activity of RMPs was assayed by thromboelastography (TEG). Pooled particle-free plasma (PFP) was obtained by centrifuging citrated fresh frozen plasma (OneBlood, Ft. Lauderdale, FL, United States) at 18,000 × *g* for 30 min to remove all cells and particles, then PFP from 3 units was pooled together to form a large batch of PFP. The batch was then aliquoted into 1.5 mL vials and was stored at -80°C, 320 μL of PFP was mixed with 10 μl of saline or RMP with 1:10, 1:20, 1:40, and 1:80 dilution for 5 min, then 20 μL of calcium was added to initiate the coagulation. A saline group was considered as control and all comparisons were made with this group. The R time (lag time) was recorded and was used for calculating the stability. The shortening of R time in the presence and absence of RMP is a measurement of procoagulant activity. The ratio of procoagulant activity at different intervals to the procoagulant activity at Day 0 is a measure of stability. The effective dose (ED) 50, and 80 were defined as below.

ED_50_ or ED_80_ = RMP concentrations required to achieve 50% or 80% of maximum reduction in “R” time, respectively.

### *In Vivo* Animal Procedures

All animal procedures were carried out on male Sprague-Dawley rats (Charles River Laboratories International, Inc., Wilmington, MA, United States) in accordance with the Guide for the Care and Use of Laboratory Animals published by the National Institutes of Health under protocols (protocol # 15-174) approved by the Animal Care and Use Committee of the University of Miami.

### *In Vivo* Pharmacokinetics

#### Preparation of Test Animal

Rats were anesthetized with 5% isoflurane in a mixture of 33% oxygen and 67% nitrous oxide. The animals were then paralyzed with rocuronium (10 mg/kg, i.v. every 20 min), artificially ventilated, and maintained on anesthesia with 1 – 2% isoflurane in a mixture of 33% oxygen and 67% nitrous oxide. An incision was made in the flank region; the femoral artery and vein were cannulated. Physiological parameters viz., body and head temperature, blood pressure, blood pH, pO_2_, and pCO_2_ were maintained in normal range before injection and then monitored until the end of the experiment. Animals showing variations in physiological parameters outside the normal range during the experiments were excluded from the present study.

#### Dose Preparation and Administration

Lyophilized RMPs stored at -80°C were reconstituted in distilled water and passed through 2 μm filters. They were briefly vortexed at room temperature and administered as per protocol explained in **Figure 5**. Dosing was carried out using an infusion pump (Stoelting Scientific Syringe Pump, Arlington, TX, United States) into the cannulated femoral vein.

#### Pharmacokinetic Sampling

The pharmacokinetics of multiple different dosing regimen were assessed as follows:

In the single bolus RMP treatment group (total dose = 6.07 × 10^10^ RMPs/kg, i.v.), 0.3 mL blood samples were obtained at 0 (pre-dose), 1, 3, 5, 6, 10, 15, 30, 45, and 60 min post-dose.

In the 1/3 bolus three times RMP treatment group, three consecutive bolus injections (each injection of 2.02 × 10^10^ RMPs/kg, i.v.) administered over 20 s contained one third of total dosage, were given after every 7.5-min interval. Blood samples of 0.3 mL each were obtained at 0 (pre-dose), 1, 3, 5, 7, 9, 11, 13, 15, 17, 19, 21, 23, and 30 min post-first dose.

In RMP dose infused over 30 min group (total dose = 6.07 × 10^10^ RMPs/kg, i.v.), one bolus injection of one-third of the total dose administered over 20 s (dose = 2.02 × 10^10^ RMPs/kg, i.v.) was followed, after a gap of 40 s, by an infusion of two-thirds of the total dose administered over the remaining 29 min (dose = 4.05 × 10^10^ RMPs/kg, i.v.). Blood samples of 0.3 mL each were obtained at 0 (pre-dose), 1, 3, 5, 10, 15, 20, 25, 30, 31, 33, 35, and 45 min post-first dose.

In the four times the single dose group, one bolus injection of one-tenth of the total dose administered over 20 s (dose = 2.43 × 10^10^ RMPs/kg, i.v.) was followed, after a gap of 40 s, by an infusion of nine–tenths of the total dose administered over the remaining 59 min (dose = 2.19 × 10^11^ RMPs/kg, i.v.). Blood samples of 0.3 mL each were obtained at 0 (pre-dose), 1, 3, 5, 10, 20, 30, 40, 50, 60, 61, 63, and 75 min post-first dose.

In the 20 times the single dose group, one bolus injection of one-tenth of the total dose administered over 20 s (dose = 1.21 × 10^11^ RMPs/kg, i.v.) was followed, after a gap of 40 s, by an infusion of nine–tenths of the total dose administered over the remaining 59 min (dose = 1.09 × 10^12^ RMPs/kg, i.v.). Blood samples of 0.3 mL each were obtained at 0 (pre-dose), 1, 3, 5, 10, 20, 30, 40, 50, 60, 61, 63, and 75 min post-first dose.

To replace the blood volume lost during sampling, an intravenous infusion of normal saline was given for the period between obtaining first and last blood samples. The total volume of saline administered through RMP injection and additional vehicle injected to compensate for bloodletting was equivalent to 3 mL/hr.

#### Pharmacokinetic Sample Processing

The blood samples collected from rats at different intervals were centrifuged at 800 × *g* for 10 min to obtain platelet poor plasma (PPP). Twenty μL of PPP was incubated with 4 μL of anti-CD235a-PE for 20 min on a shaker (60 rpm). The sample was then diluted with 976 μL of PBS and was ready for flow cytometric assay. The detailed procedures and settings were described in the previous section.

*Determination of elimination half-life (T_1/2_), volume of distribution (V_d_), elimination rate constant (k_e_), and clearance (CL):* These parameters were calculated using the following equations:

$$T_{1/2}\;\text{=}\;(t^{*}0.693)/ln(N_{0}/N_{t}),$$

Where *N*_0_ and *N*_t_ are RMP concentrations at 1 and 5 min post RMP infusion, and *t* = 4.

$$k_{e}\;\text{=}\;ln(2)/T_{1/2}$$

$$V_{d}\;\text{=}\;D/C_{0,}$$

where *D* = total RMP infused, *C*_0_ = *C*_1_/(1/2)^*t*/*T*_1/2_^

$$CL\;\text{=}\;V_{d}^{*}K_{e}$$

#### Statistics

All data points displayed in Tables and figures are mean ± SEM. Student’s *t*-test was used to compare two groups. *P* < 0.05 was considered significant.

## Results

### Quality/Purity of RMP

#### Protein

**FIGURE 1:**
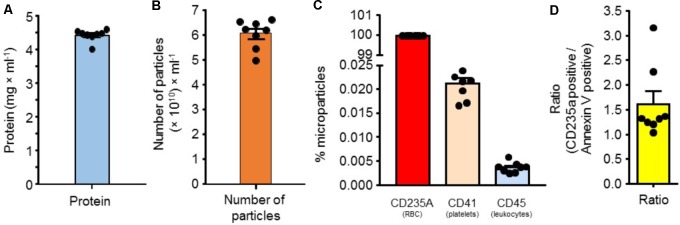
**(A)** Protein quantity in suspension of RMPs (*n* = 8). **(B)** Number of microparticles/mL of RMP in suspension (*n* = 8). **(C)** Percent of microparticles labeled with a marker for red blood cells, platelets, or leukocytes (*n* = 8). **(D)** CD235a positive/Annexin V positive particle distribution in RMPs (*n* = 8).

Protein was measured on serially diluted RMPs in normal saline. A total of eight samples were processed. We observed that the mean protein content was 4.50 ± 0.06 mg/mL of RMPs suspension (*n* = 8) (**Figure 1A**).

#### Number of Particles

We next measured the number of particles present in the above-mentioned suspension of RMPs. The number of microparticles was measured using flow cytometry as described in the methods section. Eight samples were processed. The mean density of particles was 6.07 × 10^10^ particles/mL of RMP stock suspension (*n* = 8) (**Figure 1B**).

#### Purity and Annexin V Expression

To evaluate potential contamination of RMPs from other blood cells (platelets and leukocytes), we next determined the proportion of particles labeled with markers of platelets, leukocytes, and red blood cells using CD41, CD45, and CD235a antibodies, respectively, using flow cytometry as described in the methods section. Eight samples were processed. We observed that 99.98% of particles were labeled with CD235A (RMP), while 0.021 and 0.0036% of microparticles were derived from platelets (PMP) and leukocytes (LMP), respectively. Our results demonstrate that the majority of microparticles are RMPs and only a minor portion of particles are from other cellular sources (platelets and leukocytes) (**Figure 1C**).

We next determined the mean number of annexin V-positive microparticles as a surrogate marker for phosphatidylserine. We observed this to be 3.6 × 10^10^ particles/mL of RMP stock suspension (*n* = 8). The ratio of CD235a positive/annexin V positive is around 1.6 (**Figure 1D**). This ratio of annexin V expression on RMPs indicates that these MPs possess strong pro-coagulant activity as phosphatidylserine (a procoagulant phospholipid) is mostly present in the inner leaflet of RBC membrane.

#### Size of Particles

**FIGURE 2:**
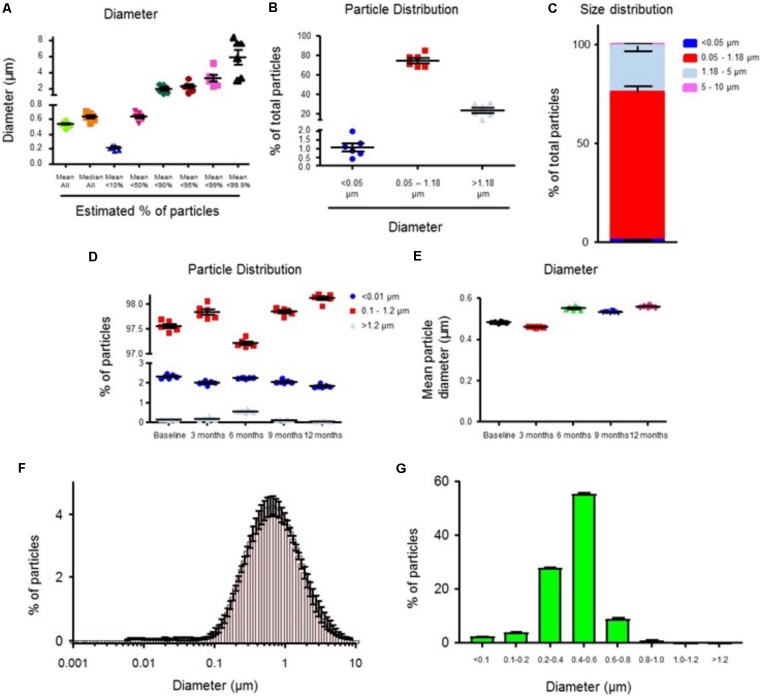
The size distribution of RMPs when analyzed using DELSA^TM^ Nano analyzer **(A–C)** and flow cytometry **(D,E)** methods. **(A)** Mean (all), median (all), and maximum diameter of RMP populations (smallest 10, 50, 90, 95, 99, and 99.9%) (*n* = 6). **(B)** Particle size distribution as a percent of total size (<0.05 μm, 0.05 – 1.18 μm, >1.18 μm) (*n* = 6). **(C)** Percent of particle distribution in the four size groups (<0.05 μm, 0.05 – 1.18 μm, 1.18 – 5 μm, and 5 – 10 μm) (*n* = 6). **(D)** The size distribution (% of total) of RMPs when analyzed using flow cytometry (*n* = 6). **(E)** Mean particle diameter (μm) in samples stored at –20°C for the duration of 0 (*n* = 6), 3 (*n* = 6), 6 (*n* = 6), 9 (*n* = 6), and 12 months (*n* = 6). The size distribution curves of RMPs when analyzed using DELSA^TM^ Nano analyzer (**F**, *n* = 6) and flow cytometry methods (**G**, *n* = 6).

We used two different methods to analyze particle size. First, we analyzed particle size using the Doppler electrophoretic light scattering analysis (DELSA^TM^ Nano) method. The mean and median diameter of RMPs was 0.54 and 0.64 μm (*n* = 6), respectively (**Figure 2A**). We observed that the size of 75% (*n* = 6) of particles was between 0.05 and 1.18 μm (**Figure 2B**). The diameter of only 0.87% (*n* = 6) of particles was between 5 and 10 μm (**Figure 2C**). The size distribution curve is presented in **Figure 2F**. These results demonstrate that the diameter of the majority of particles is between 0.05 and 1.8 μm, with a very small percent of particle diameters between 5 and 10 μm.

We also measured particle size employing flow cytometry using megamix beads as standards. A plot of log FS vs. Log (bead size) resulted in a linear regression of *y* = 2.0031*x* + 1.198, *R*^2^ = 0.9938. Based on this equation, we converted the mean FS of RMP into the mean size of RMP. We observed that 2% (*n* = 6) and 0.46% (*n* = 6) of particles were smaller than 0.1 μm or bigger than 1.2 μm, respectively (**Figure 2D**). The size of most of the particles (98%, *n* = 6) was between 0.1 and 1.2 μm (**Figure 2D**). The size distribution curve is presented in **Figure 2G**. The mean and median diameter of RMPs was 0.53 μm (**Figure 2E**) and 0.46 μm (*n* = 6), respectively. We conclude that considering the size of the particles, measured using two different methods, RMPs should not interfere with blood flow at the capillary level.

### *In Vitro* Coagulant Activity

**FIGURE 3:**
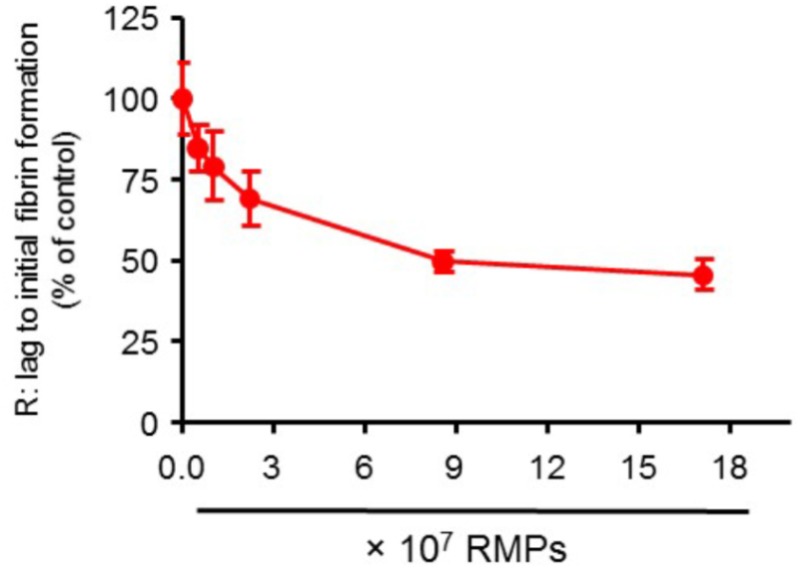
Dose response curve showing the effect of variable RMP counts on R: lag time to initial fibrin formation. *N* = 20, 9, 8, 10, 10, and 20 for zero, 0.51 × 10^7^, 1.03 × 10^7^, 2.23 × 10^7^, 8.57 × 10^7^, and 17.1 × 10^7^ particles/mL groups, respectively. Results are presented as mean ± SEM.

*In vitro* coagulant activity was measured by thromboelastography (TEG) as described previously (Jy et al., 2013). The desired number of RMPs were added to PFP and incubated at room temperature for a minute before adding CaCl_2_. Lag time to initial fibrin formation in minutes (*R*) was measured using TEG and ED_50_ and ED_80_ values were calculated from the dose-response curve (**Figure 3**). We observed a reduction in lag time, *R*, with increasing concentrations of RMPs. The *R* time in the presence of zero RMP concentration (control group) was 29.51 min. The *R* in the presence of zero, 0.51 × 10^7^, 1.03 × 10^7^, 2.23 × 10^7^, 8.57 × 10^7^, and 17.1 × 10^7^ particles was 100 (*n* = 20), 85 (*n* = 9), 79 (*n* = 8), 69 (*n* = 10), 50 (*n* = 10), and 46% (*n* = 20) of control, respectively. We observed ED_50_ and ED_80_ of 1.7 × 10^7^ and 6.0 × 10^7^ particles/mL, respectively. Our results demonstrate the ability of RMPs to accelerate fibrin formation time *in vitro*.

### Stability/Shelf-Life

To ascertain both patient safety and management of the RMPs, we determined the shelf-life (expiry date) at five different storage conditions [40°C + 75% relative humidity (RH); 30°C + 65% RH; 25°C + 60% RH; 5°C; and -20°C] under three study arms (long-term, intermediate, and accelerated). The effect of storage conditions on physical properties of RMPs is presented in **Table 1**. For baseline control group, the reduction in “*R*” time was 13.85 ± 1.37 min. For the 3-month storage duration, upon return, RMPs in vials appeared similar to baseline vials for -20°C, and 5°C storage conditions. However, the color appeared dull pink to brownish for 25, 30, and 40°C storage conditions.

**Table 1:** Effect of storage conditions on physical properties of RMPs.

Storage condition	Storage duration (months)	Physical properties				
		Appearance	Color	Texture	Solubility	Odor
**-20°C**	3	Dry powder pellet	Bright pink	Powder	Easy to dissolve	No odor
	6	Dry	Bright pink	Powder	Easy to dissolve	Mild odor
	9	Dry	Pink	Powder	Easy to dissolve	Mild odor
	12	Dry	Pink	Powder	Easy to dissolve	Mild odor
**5°C**	3	Dry powder pellet	Bright pink	Powder	Easy to dissolve	No odor
	6	Dry	Bright pink	Powder	Easy to dissolve	Mild odor
	9	Dry	Pink	Powder	Easy to dissolve	Mild odor
	12	Dry	Pink	Powder	Easy to dissolve	Mild odor
**25°C + 60% RH**	3	Dry powder pellet	Dull pink	Powder	Easy to dissolve	No odor
	6	Dry	Dull pink	Powder	Easy to dissolve	Mild odor
	9	Dry	Brownish	Powder	Easy to dissolve	Mild odor
	12	Dry	Brownish	Powder	Easy to dissolve	Mild odor
**30°C + 65% RH**	3	Dry powder pellet	Darker pink	Powder	Easy to dissolve	No odor
	6	Dry	Brownish	Powder	Easy to dissolve	Mild odor
**40°C + 75% RH**	3	Dry powder pellet	Brownish	Powder	Difficult to dissolve	No odor
	6	Dry	Brownish	Powder	Difficult to dissolve	No odor

*RH, Relative humidity.*

**FIGURE 4:**
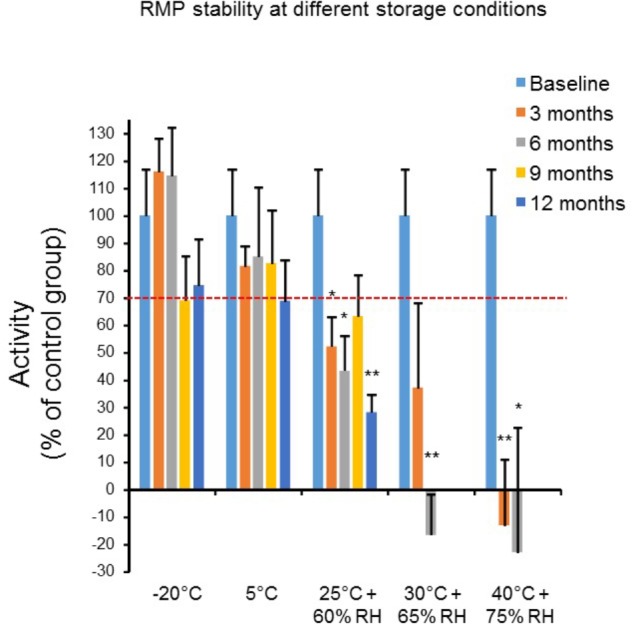
The effect of different storage conditions (40°C + 75% relative humidity: RH, *n* = 6; 30°C + 65% RH, *n* = 6; 25°C + 60% RH, *n* = 6; 5°C; and -20°C, *n* = 6) and duration (baseline, 3, 6, 9, and 12 months) on *in vitro* procoagulant activity (R: lag time to initial fibrin formation). The lag time for each storage condition is expressed as a percent of control group. Results are presented as mean ± SEM. ^∗^*p* < 0.05, ^∗∗^*p* < 0.005 vs. baseline.

The effect of storage conditions on the procoagulant activity of RMPs is shown in **Figure 4**. At -20°C, the activity remained about 70% of baseline activity for at least 12 months (six observations for baseline and all storage conditions). At 5°C, the activity remained above 80% of baseline for 9 months (six observations for baseline and all storage conditions). At other conditions (40°C + 75% RH: six observations for baseline and all storage conditions, 30°C + 65% RH: six observations for baseline and 3 months storage group and four observations for 6 months storage group, and 25°C + 60% RH: six observations for baseline and all storage conditions), the activity declined rapidly below 80% after 3 months of storage.

**FIGURE 5:**
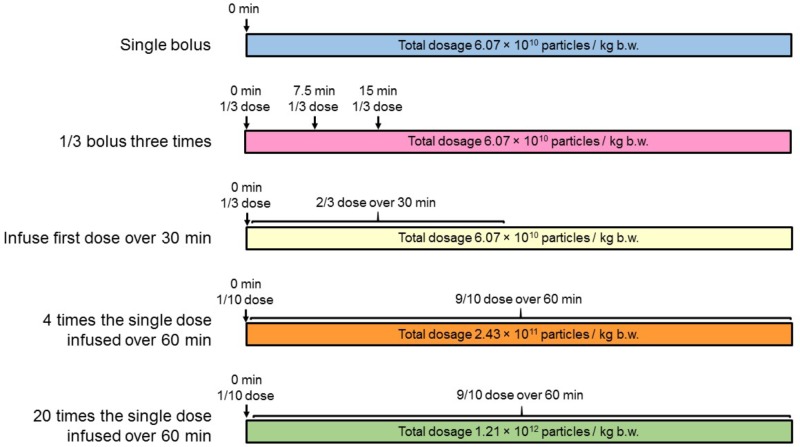
Schematic illustrating the treatment regimens used. (1) A single bolus (*n* = 10), (2) 1/3 bolus three times (*n* = 10), (3) infuse first bolus dose over 30 min (*n* = 10), (4) four times the single dose infused over 60 min (*n* = 10), and (5) 20 times the single dose infused over 60 min (*n* = 10).

### *In Vivo* Pharmacokinetic Profile of RMPs

**FIGURE 6:**
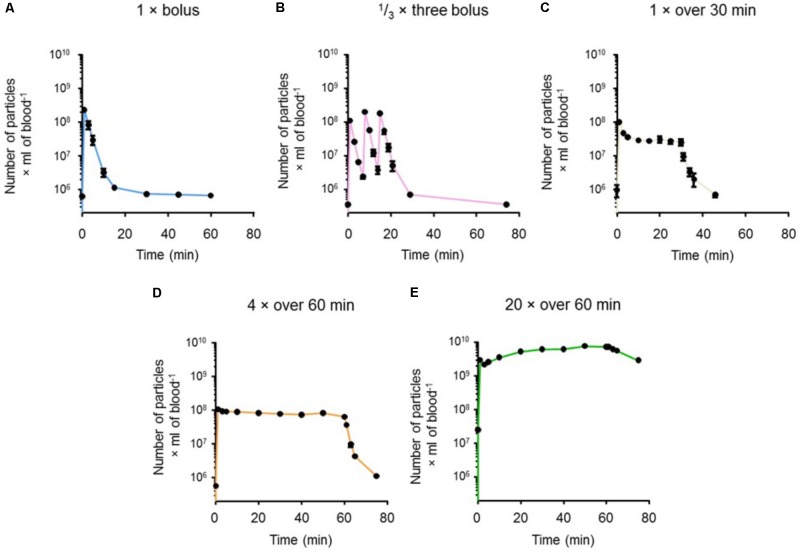
Curves showing RMP levels in blood as measured by flow cytometry for **(A)** single bolus (*n* = 10), **(B)** 1/3 single bolus 3 times (*n* = 10), **(C)** a single bolus infused over 30 min (*n* = 10), **(D)** four times the single dose infused over 60 min (*n* = 10), and **(E)** twenty times the single bolus infused over 60 min (*n* = 10). Results are presented as mean ± SEM.

**FIGURE 7:**
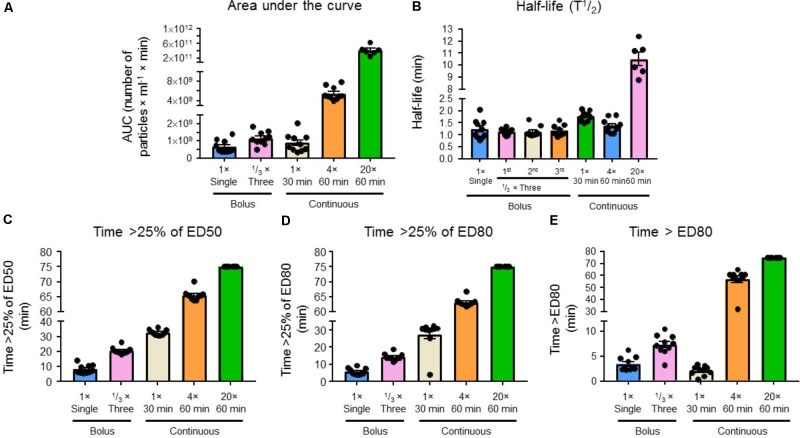
**(A)** Area under the curve, **(B)** half-life (T½), **(C)** time >25% of ED_50_, **(D)** time >25% of ED_80_, and **(E)** time > ED_80_ for a single bolus (*n* = 10), 1/3 bolus three times (*n* = 10), infuse first bolus dose over 30 min (*n* = 10), four times the single dose infused over 60 min (*n* = 10), and 20 times the single dose infused over 60 min regimens (*n* = 10). Levels of RMPs were measured by flow cytometry. Results are presented as mean ± SEM.

Levels of RMPs in blood over time for the five experimental regimens are presented in **Figure 6**. We achieved blood plasma concentrations of RMPs ≥ 25% of the target therapeutic concentration (ED_80_ = 6 × 10^7^ particles/mL), as inferred from TEG data, in all five treatment regimens (**Figure 7D**). **Figure 7C** shows the times at which blood RMP levels of >25% of ED_50_ was achieved for 1 × bolus (*n* = 10), 1/3 × three bolus (*n* = 10), 1 × over 30 min (*n* = 10), 4 × over 60 min (*n* = 10), and 20 × over 60 min (*n* = 6) groups; these times were 8, 21, 33, 66, and 75 min, respectively. **Figure 7D** shows the times at which >25% of the target *in vitro* ED_80_ was achieved for 1 × bolus (*n* = 10), 1/3 × three bolus (*n* = 10), 1 × over 30 min (*n* = 10), 4 × over 60 min (*n* = 10), and 20 × over 60 min (*n* = 6) groups; these times were 6, 14, 28, 63, and 75 min, respectively. **Figure 7E** shows the total time for which blood RMP levels above ED_80_ levels.

**FIGURE 8:**
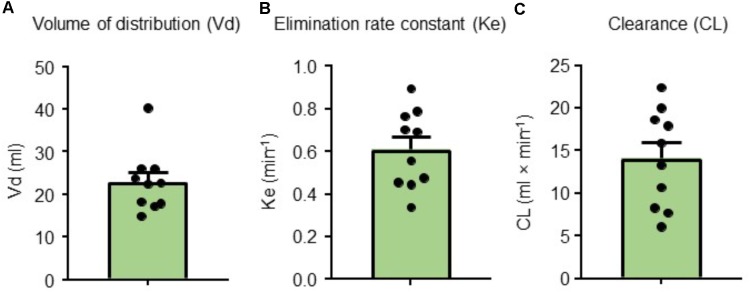
**(A)** Volume of distribution (*V*_d_), **(B)** elimination rate constant (*K*_e_), and **(C)** clearance (CL) values for a single bolus group (*n* = 10). Results are presented as mean ± SEM.

The area under the curve for each group (1 × bolus: *n* = 10, 1/3 × three bolus: *n* = 10, 1 × over 30 min: *n* = 10, 4 × over 60 min: *n* = 10, and 20 × over 60 min: *n* = 6) is presented in **Figure 7A**. The time required (half-life) to reach half-dose from the end of injection in a single bolus (*n* = 10), infused first bolus dose over 30 min (*n* = 10), four times the single dose infused over 60 min (*n* = 10), and 20 times the single dose infused over 60 min (*n* = 6) was 1.24, 1.78, 1.38, and 10.50 min, respectively (**Figure 7B**). The half-life from the end of the first, second, and third injection for 1/3 × three bolus group (*n* = 6) was 1.14, 1.13, and 1.18 min, respectively. For the single bolus group (*n* = 10), we also calculated the volume of distribution (*V*_d_) (23 mL), elimination rate constant (*K*_e_) (0.61/min), and clearance (CL) (14.09 mL/min) (**Figure 8**). Overall, our results indicate that the 4 × over 60 min regimens appears optimal as this regimen is able to maintain blood levels of RMPs relatively stable during infusion duration.

## Discussion

Recent studies have explored the hemostatic properties of RMPs and their potential use as therapeutic agents in bleeding disorders. Procoagulant properties of RMPs have been documented including their role in the coagulation cascade, thrombin generation, and acceleration of fibrin formation (Van Der Meijden et al., 2012; Jy et al., 2013; Rubin et al., 2013). Our experimental evidence supports earlier findings demonstrating the hemostatic properties of RMPs and expands on the pharmacokinetic analysis of these particles in addition to their suitability in future therapeutic administration for bleeding disorders.

In addition to RMPs, other cell-derived microparticles have previously been evaluated as hemostatic agents, each accompanied by side-effects. Platelet derived MPs (PMPs) express tissue factors, therefore possess increased risk of thrombosis. PMPs have a number of additional effects; researchers have found that PMPs induce cyclooxygenase-2 production by endothelial cells (Barry et al., 1997) causing subsequent platelet activation. PMPs also release cytokines, contributing to their proinflammatory and proatherogenic properties (Mause et al., 2005). Additionally, leukocyte derived MPs (LMPs) have inflammatory and atherosclerotic properties and have been linked to vascular injury (He et al., 2017). In contrast to the side-effects of PMPs and LMPs, RMPs cause fewer unwanted adverse events (Jy et al., 2013), such that transfusion of RMPs will have the intended hemostatic effects without causing the proinflammatory effects that other microparticles instigate. The fact that RMPs lack tissue factors also highlights lower risk of thrombosis (Jy et al., 2013). This is supported by the well-documented evidence that cell-derived MPs have the same properties and safety profile as their parent cells (Leroyer et al., 2007; Gelderman and Simak, 2008; Burnier et al., 2009). In light of the transfusion complications with other types of cell-derived MPs, sample purity is essential to confirm the safety profile of an infusion. Our results indicate successful isolation of pure RMPs (**Figure 1C**). This purity and the likeness of RMP properties to their parent cells indicate that an infusion with RMPs will have a similar safety profile to a transfusion of RBCs.

The PS is expressed on the inner membrane leaflet and becomes externalized when RBCs shed microparticles (Burnier et al., 2009). We used a high pressure extrusion method to prepare RMPs. It is possible that variable amount of red blood cell membrane fragments may seal either inside-out or right side-out during this process. Since the inner leaflet of red blood cell membranes is rich in phosphatidylserine (PS), the ratio of inside-out and right side-out particles may determine the procoagulant activity of RMPs (Burnier et al., 2009). One possible way to establish this ratio is by determining presence of annexin V reactivity (a PS proxy). This allows for the membrane orientation of particles to be determined. Thus, by comparing annexin V-positive MPs to those expressing CD235a (a marker for particles that have not undergone inversion of their membrane leaflets), we determined the ratio of MPs that had hemostatic properties and would contribute to activation of coagulation cascade (Leventis and Grinstein, 2010). We found this ratio to be 1.6 (**Figure 1D**). This ratio of annexin V positive MPs to those expressing CD235a could be used for quality control as a marker of purity in future preparation for therapeutic administration of RMPs. An earlier study demonstrated that RMPs may enhance procoagulant activity via the extrinsic pathway of coagulation (tissue factor) (Fischer et al., 2017). Here, the levels of tissue factor on monocytes increased following its incubation with RMPs for 2 h. Since we observed a short half-life for RMPs *in vivo*, it is plausible that the tissue factor-dependent pathway may not play a significant role in RMP-induced enhanced procoagulant activity *in vivo*. In an earlier study we did not observe the presence of tissue factor on RMPs prepared by high pressure extrusion method (Jy et al., 2013). In view of this result, we did not determine the level of tissue factor in our preparation.

To further ensure the safety of administered RMPs, the additional analysis of particle diameter was investigated in our present study (**Figure 2**). Particle diameter was necessarily assessed, as the size of therapeutic agents given systemically must be gauged to confirm that they will not occlude blood vessels or impede blood flow. Previous studies have shown that size of RMPs ranges between 0.1 and 1 μm (Owens and Mackman, 2011). Our size estimate methods revealed RMPs to have a mean diameter of <0.6 μm, with most of the particles between 0.05 and 1.18 μm. Other systemically injected biomaterial therapies have a wide range of diameters, including: polymeric nanoparticles used for drug delivery across the BBB (0.01–1 μm) (Orive et al., 2009), platelets (∼2 μm) (Freitas, 1999), glial-restricted precursors (15 μm) (Janowski et al., 2013), mesenchymal stem cells (25 μm) (Janowski et al., 2013), and hematopoietic stem cells (∼30–40 μm) (Freitas, 1999). Given the use of these systemically injected biomaterial therapies, the size of RMPs identified in this study indicates that they would also be safe systemically injectable therapies, and would not increase the risk of blocking vessels or reducing blood flow to tissues.

Equally important to the safety profile, we investigated the effects that RMPs elicit *in vitro* and *in vivo*. We found an *in vitro* dose-response relationship between the number of RMPs and their procoagulant activity *in vitro* (**Figure 3**). A steady elevation in the coagulative effect was observed as the concentration of RMPs was augmented until a threshold was reached, at which point a saturating effect was observed at additionally higher doses of RMPs. This is possibly due to a fixed number of sites where RMPs can bind, and beyond this saturation concentration, all binding sites are occupied. We thus hypothesize that there is an optimal ratio at which the maximal hemostatic therapeutic benefit from RMPs can be achieved. It should be also noted that RMPs does not possess all components of coagulation cascade. However, they act as a catalyst, increasing reaction time without actually participating in the hemostasis. Since number of platelets are higher in our experimental conditions compared to number of RMPs, it is also possible that some other mechanisms may be responsible for saturating effect that we are observing.

Our *in vivo* experiments provided additional support to this hypothesis of a finite number of RMP binding sites. By evaluating different paradigms, we assessed various bolus and maintenance dose combinations; in one of the investigated paradigms we were able to maintain levels with continuous infusion (20 × over 60 min regimen) without reaching a plateau (**Figure 6E**). This provides evidence that RMPs bind to a fixed number of available sites. Once these sites are saturated, the RMPs remain unable to bind in the blood, increasing RMP concentration in the serum. However, this hypothesis remains to be tested. Our findings additionally indicate that employing different paradigms could allow for RMPs to be advantageous as hemostatic agents in a number of settings depending on the particular bleeding profile. During surgery, continuous infusion would expectedly be appropriate, whereas in hemorrhagic stroke, a bolus to reach binding saturation and then maintenance doses would be more effective. The half-life of RMPs determined in our *in vivo* experiments also substantiates the suitability of these particles as a hemostatic agent (**Figure 7A**). Our *in vivo* experiments showed the half-life of RMPs to be very short, averaging <90 s. An earlier study by Willekens et al. (2005) studied clearance of rat red blood cell-derived vesicles in Wistar rats. This earlier study evaluated the effects of naturally occurring vesicles as blood cell vesicles were isolated from rat plasma. They observed that 80% of particles were removed from the circulation within 5 min of administration. This study further supports our observations. It remains to be tested if short half-life also translates into short lasting effects on its hemostatic properties. While additional studies are needed to determine the potential for wide spread use of RMPs, our results demonstrate that they have the potential to be used as a hemostatic agent in many bleeding disorders including hemorrhagic stroke. Pharmacokinetic parameters computed from the plasma concentration time data viz., elimination rate constant, clearance, and volume of distribution may help in designing dosing regimens for disease conditions requiring a steady state concentration of RMPs for long-term treatment. Prior to clinical assessment, information about disposition and clearance of RMPs in animal models may help in predicting margins of safety based upon exposure and dose (ICH, 1997).

To determine the long-term suitability of these particles for their intended use, the pharmaceutical stability was evaluated, which afforded sufficient evidence that RMPs will retain an appropriate quality throughout the marketed period and the suitability for therapeutic administration will be preserved (Bajaj et al., 2012). The outcome of these evaluations showed that RMPs are stable for 12 months, meeting ICH guidelines (Bajaj et al., 2012). Implications of these stability tests indicate that RMPs will maintain their integrity, efficacy, and safety throughout the course of the expected shelf life of 1 year. We used O Rh+ blood to prepare RMPs. We did not evaluate levels of minor antigens during preparation of RMPs. Considering potential immune responses of Rh antigen and other minor antigens, detailed safety studies are warranted before its use in the clinic.

## Conclusion

We are the first to report comprehensive pharmacokinetics for red blood cell microparticles. Our study will serve as a guideline for future pharmacokinetic studies for other types of microparticles. Our pharmacokinetic results will also provide a strong foundation for designing treatment regimens for RMPs to be used as a therapeutic agent in various bleeding conditions. Our results demonstrate that RMPs derived from human blood are stable during prolonged storage and are amenable to various dosing regimens for treating diseases involving abnormal blood coagulation. Nevertheless, further studies are required to characterize safety, toxicology, efficacy, and disposition prior to realizing the clinical potential of RMPs as a treatment strategy.

## Author Contributions

WJ and KD designed the experiments. AR, CB, and HN-Q performed the experiments. AR, CB, WJ, and KD analyzed the data. WJ, AR, CH, and KD wrote parts of the manuscript. SK and YA provided clinical view points while designing experiments. All authors critically reviewed the manuscript.

## Conflict of Interest Statement

RxMP Therapeutics provided the testing material for the study. YA, WJ, and the University of Miami have partial ownership in RxMP Therapeutics. YA and WJ also received a grant support from RxMP Therapeutics. The remaining authors declare that the research was conducted in the absence of any commercial or financial relationships that could be construed as a potential conflict of interest.
